# Knowledge of Infection Prevention and Control and Practice Behaviors among Career and Volunteer Firefighters in Rural Communities

**DOI:** 10.3390/merits4020011

**Published:** 2024-04-10

**Authors:** Sanyang Edrisa, Adams Ashley, Taylor Ritchie, McDonalds Vernell, Macy Gretchen, Basham Jacqueline

**Affiliations:** 1 Center for Environmental and Workplace Health, Department of Public Health, College of Health and Human Services, Western Kentucky University, Bowling Green, KY 42101, USA; 2 Environmental and Occupational Health Graduate Program, Department of Public Health, College of Health and Human Services, Western Kentucky University, Bowling Green, KY 42101, USA

**Keywords:** infection prevention and control, firefighters, career, volunteer, dayroom, in-vehicle

## Abstract

Due to the emerging threat conditions in the work environment, firefighters are at a high risk of exposure to not only toxic substances but also biological agents in the dayroom and during emergency runs. The aim of this study is to evaluate firefighter (career and volunteer) knowledge and practice behaviors on infection control. This study surveyed 444 firefighters (210 career, 234 volunteer) in rural Northwestern Kentucky. The self-reported survey focused on individual characteristics, knowledge on exposure incident control, precautionary actions, and personal protections. We evaluated the descriptive characteristics of knowledge and practice scores stratified by firefighter groups (career and volunteers). The associations between infection control training received (yes/no) and firefighter knowledge and practice scores were also examined. Firefighters who were trained on infection control prevention had significantly higher knowledge scores (M = 63.7, SD = 13.4 vs. M = 59.7, SD = 15.9; *p* = 0.012). Volunteer firefighters exhibited better infection control practice behaviors than career firefighters (M = 70.6, SD = 13.0 vs. M = 67.4, SD = 11.1; *p* = 0.05). Firefighters who followed infection control guidelines (M = 69.5, SD = 11.9 vs. M = 58.1, SD = 9.9; *p* = 0.012) and expressed need for a comprehensive training on personal protective equipment (PPE) selection (β = 3.41, SE = 1.54, aOR = 30.22, 95% CI: 1.47–620.87; *p* = 0.028) had significantly higher practice scores compared to those who did not. The study results have policy implications for infection prevention and control (IPC) in rural fire departments, both career and volunteer. A review of infection control policies is needed, especially as it relates to training and practice behaviors during emergency calls and in the dayroom. Results also suggest the need to develop strategies to improve the culture of PPE use and training on the selection of PPEs appropriate to the emergency response type.

## 1. Introduction

Firefighters are faced with numerous risks and health hazards while in the dayroom and during emergency responses. In this study, dayroom was categorized as the common room/space where firefighters interact, relax, or perform other activities while waiting for an emergency. As first responders, the nature of firefighters’ work often puts them in situations where they respond to emergency calls with incomplete information [1,2]. The response situations are often complicated because response activities are provided in heterogeneous and uncontrolled circumstances. First responders have been further challenged during the COVID-19 pandemic. Throughout the pandemic, first responders have been found to experience high levels of fatigue, reduced motivation and productivity, and increased risks of exposure to both chemical contaminants and infectious agents [3]. Because of these and other factors, the firefighter occupation has been ranked among the top high-risk occupations in the United States [4,5].

During fire or emergency medical service response calls, information provided to responders is usually limited [3]. Infectious disease status of the patients is not always known. As a result, firefighters are in frequent contact with high-risk populations, including engaging with patients in uncontrolled environments [2,6]. While on-duty firefighter fatalities have declined by 27% between 2011 and 2020 [7], exposure to biologic contaminants was the second leading cause of death in 2020 [7]. Firefighters’ exposures to infectious agents are increased in work environments that may include emergency response vehicles or in the dayroom. These multiple exposure sources could lead to worker and patient safety issues [7,8].

The World Health Organization (WHO) and Center for Disease Control and Prevention (CDC) consider infection prevention and control (IPC) as a practical and evidence-based approach, which helps to prevent patients and first responders from harmful infections. In daily firefighter operations, the potential for transmission of infectious agents is possible in the dayroom and during runs [2,9]. They are exposed to viruses, bacteria, and other infectious agents through contact with blood and other bodily fluids [2,9]. Firefighters can also be exposed to biological contaminants such as molds, mildews, and dust mites in the indoor air in the dayroom and other workspaces. It is extremely important to protect these high-risk workers against biological contaminants. Effective methods of IPC include continuous action at all levels of emergency response systems [4,10,11]. Firefighter knowledge on transmission pathways of biologic contaminants, precautionary actions needed to minimize exposure, and practice behaviors that promote efficient service provision is critical. Additionally, it is also equally important to encourage a practice of behaviors that minimizes potential for cross contamination between the responder and the patient. Knowledge and practice behaviors on the use of personal protective equipment (PPE) for different response activities and exposure scenarios are especially important [2,10,11]. Lack of knowledge and low practice behaviors on IPC combined with an unawareness of prevention during response and in the dayroom and the potential risks of transmission of contaminants to patients (and co-workers) constitute a barrier to IPC compliance. To overcome this barrier, improved knowledge and practice behaviors through continuing education and training are fundamental to improve IPC practices [12,13].

Western Kentucky University (WKU) Center for Environmental and Workplace Health (CEWH) research team previously identified a concern for negative health outcomes with firefighter exposures to work-related toxic substances and behaviors on management of turnout gears (coats and pants) [13–15]. Overall, there is limited literature on firefighters’ exposure to biological contaminants; most of the previous studies are limited to chemical exposures [14–16] and were conducted in urban areas [17–19]. Therefore, the purpose of this study was to compare career and volunteer firefighters’ knowledge and practice behaviors on infection transmission in the dayroom and during emergency runs. A secondary aim is to explore opportunities to improve training in the broad discipline of occupational health for rural firefighters.

## 2. Materials and Methods

Human Subjects Institutional Review Board (IRB) approval for this study was issued by Western Kentucky University, Protocol Number: 21-096.

### 2.1. Study Population

This study included 444 career and volunteer firefighters in the Green River Firefighters Association (GRFA), encompassing 70 career and volunteer fire stations in a 10-county region. GRFA is the largest career and volunteer firefighter organization in Kentucky. Members meet bi-monthly to discuss the profession as well as to share best practices and priority issues for their health and wellbeing. Various training needs are also discussed during the bi-monthly meetings. Members of the WKU CEWH attend these meetings and have a standing agenda item to discuss current participatory research with GRFA. This supports further development of a community-based participatory research model with rural firefighters [15].

### 2.2. Survey

A survey titled “Firefighter practices and exposures in response to emergency calls in Northwestern Kentucky” was developed to identify gaps in knowledge and practice and to prioritize occupational health concerns with respect to exposure to biologic agents. Personal contacts by the project team were established by attending bi-monthly GRFA monthly meetings and exchanging ideas on research and training needs in occupational safety and health. The survey included a broad range of questions on issues including knowledge on exposure incident control, infection control practice, knowledge on precautionary actions, knowledge on personal protection, and infection control during COVID-19 response. Due to the rurality of the region, the survey was distributed in person, by the project graduate research assistant, to all career and volunteer fire stations in the region. The survey was administered in an exam-like setting during one of the individual fire station weekly meetings. The project graduate assistant, a volunteer firefighter, was present in all the meetings when the surveys were completed to ensure independence and control cheating. Study participants completed the survey using paper and pencil, which were later entered in Qualtrics^®XM^ for data management.

### 2.3. Data Analysis

The survey was developed in English and self-administered during one of the individual fire departments weekly meetings. Participants were asked to respond to knowledge tests on infection control in the dayroom and during emergency responses. The knowledge and practice test items included true/false, select the best answer, and rank-order questions. Responses were collected over a period of eight months, and for the purpose of analysis, missing responses were coded as “no knowledge or practice activity”. Analysis, including calculation of frequencies and distribution of survey responses by participants’ characteristics, was completed using SAS 9.4 (SAS Institute, Inc., Cary, NC, USA).

The survey comprised 34 questions, which were organized into four main sections. The first section, titled “Sociodemographic Characteristics”, contained six questions. The second section, “Knowledge on Exposure Incident Control”, consisted of eight questions and is detailed in Table A1 (Appendix A). The third section, “Infection Control Practices Towards COVID-19 Response”, had ten questions and is also detailed in Table A1 (Appendix A). The fourth and final section, “Actual Infection Control During COVID-19 Response”, contained ten questions. Both the “Knowledge on Exposure Incident Control” and “Infection Control Practices” sections had a maximum total score of 12 and 18, respectively. The scores were converted into cumulative percentages and served as a continuous outcome variable for analysis in this paper. The indicators used to assess COVID-19 KP were based on lessons learned from similar studies in the context of infectious diseases (Table 1).

During analysis, the focus was on 13 variables: age, gender, firefighter group, years of experience, firefighter status (career or volunteer), compliance with infection prevention guidelines, use of PPE during response to emergency calls, types of PPE worn during COVID-19 responses, if the firefighter responded to COVID-19 calls while on the job, if the firefighter is trained on infection prevention, topics to be addressed when training firefighters on PPE selection, and if participants tested positive for COVID-19. All these variables were factored in to determine the knowledge and practice behaviors of firefighters surveyed. Based on the age distribution and objective to examine the entire working profession, age was collapsed according to the decade of life: <30 (24.5%), 30–40 (33.6%), and >40 years (41.9%). Topics to be addressed when training firefighters on PPE selection include how to protect against self-contamination, safe removal of disposal gloves, how and when to wear PPE, and what to do in an event of equipment failure. Responders selected which of the topic areas they feel need training on. The topic areas were collapsed as indicated in Table 1.

Distribution of the primary variables were examined by firefighter status (career and volunteer) to identify general characteristics and trends using Chi-square tests. Unpaired *t*-test, One-way Analysis of Variance (ANOVA), as well as Generalized Linear Model (GLM) for classical regressions were used to identify associations between firefighter practices and knowledge using various factors related to infection control. To assess the independence of the 13 selected covariates, a multicollinearity test was performed using a variance inflation factor and tolerance level thresholds. This was conducted prior to their inclusion in the model. The model was used to estimate the adjusted odds ratio (aOR); a 95% confidence interval and a *p*-value < 0.05 were used to determine statistically significant associations.

## 3. Results

### 3.1. General Characteristics

In the comparative analysis of 444 firefighters, composed of 210 career and 234 volunteer participants, significant differences were observed across multiple demographic and professional variables (Table 2). The sex distribution was notably disparate, with a higher prevalence of females in the volunteer group (13.7%) than in the career group (2.4%) at *p* = 0.001. Variations were also evident in firefighter roles (*p* = 0.028), with Fire Chiefs and those with over 20 years of experience being more common among volunteers. The age distribution further differed significantly (*p* = 0.001), with a higher proportion of career firefighters falling in the 30–40 years age bracket, while volunteers were more frequently above 40 years. Years of experience also showed a significant difference (*p* = 0.001), with career firefighters being more likely to have 11–20 years of experience, whereas volunteers had more than 20 years of experience. Overall, knowledge and practice scores on IP were low. The highest mean for knowledge and practice scores were 71.9 and 70.9 respectively, which were lower than expected (100%).

### 3.2. Knowledge Dimension Related to IPC

In the evaluation of firefighters’ knowledge of IPC, several variables were statistically significant (Table 3). Gender was a significant factor, with females scoring higher (M = 66.7, SD = 11.4) than males (M = 62.5, SD = 14.2; *p* = 0.039). Firefighter status did not significantly influence knowledge scores (*p* = 0.639), with career firefighters (M = 62.5, SD = 13.9) and volunteers (M = 63.1, SD = 14.2) showing comparable performance. Among firefighter groups, the “Others” category had the highest mean score (M = 71.9, SD = 9.1) and the difference was statistically significant (*p* = 0.015). Firefighters who had received training on infection prevention scored higher (M = 63.7, SD = 13.4) than those who had not (M = 59.7, SD = 15.9; *p* = 0.012). Finally, comprehensive training on PPE selection was associated with the highest knowledge scores (M = 64.8, SD = 14.0), and the difference was highly significant (*p* < 0.001). To summarize, knowledge on IP was higher among firefighters categorized as needing comprehensive training, females, or as the other group.

### 3.3. Practice Dimension Related to IPC

Regarding practices related to IPC, firefighter status was a significant variable (*p* = 0.005; Table 3). Volunteer firefighters scored higher (M = 70.6, SD = 13.0) than career firefighters (M = 67.4, SD = 11.1). Years of experience also showed a significant difference (*p* = 0.033) for firefighters with 10 years or less of experience scoring the highest (M = 70.9, SD = 11.9). Firefighters who reported following infection prevention guidelines had significantly higher practice scores (M = 69.5, SD = 11.9) than those who did not (M = 58.1, SD = 9.9; *p* = 0.012). However, the use of PPE during emergency calls and the types of PPE worn during COVID-19 responses did not significantly influence the practice scores (*p* = 0.313). In summary, IP practice behavior score was higher among volunteer firefighters, those having less than 10 years of experience, or those who reported to follow infection prevention guidelines.

### 3.4. Knowledge Dimension and Its Associated Factors

In the multivariable analysis assessing the association between firefighter knowledge and various factors related to infection control, the intercept was highly significant (β = 63.83, SE = 1.60, aOR = 5.24, 95% CI: 2.28–1.20, *p* < 0.001) (Table 4). Among the variables, only training in infection prevention (IP) emerged as statistically significant. Firefighters who had not received infection prevention training had significantly lower knowledge scores (β = −3.65, SE = 1.79, aOR = 0.02, 95% CI: 7.78–0.86, *p* = 0.04). Firefighter status, gender, and age did not show a statistically significant association with knowledge scores. For instance, volunteer firefighters had an aOR of 6.00 (95% CI: 0.31–115.12, *p* = 0.235) compared to career firefighters, which means volunteer firefighters are six-times more likely to have knowledge on IP than career firefighters. This association is not significant. Similarly, females had an aOR of 66.22 (95% CI: 0.44–9895.03, *p* = 0.101) compared to males, implying that females are 66.22-times more likely than their male counterparts to have knowledge on IP. However, this association is not significant.

### 3.5. Practice Dimension and Its Associated Factors

In the multivariable analysis assessing the association between firefighter practices and various factors related to infection control, the intercept was highly significant (β = 63.89, SE = 2.50, aOR = 5.61, 95% CI: 4.16–7.55, *p* < 0.001; Table 5). Volunteer firefighters exhibited significantly better practices than career firefighters, with an aOR of 92.05 (95% CI: 8.59–986.25, *p* < 0.001). Firefighters who did not follow the infection control guidelines reported significantly worse practices (β = −9.27, SE = 4.46, aOR = 9.46, 95% CI: 1.53–0.58, *p* = 0.038). Comprehensive training in PPE selection was associated with significantly better reported practices (β = 3.41, SE = 1.54, aOR = 30.22, 95% CI: 1.47–620.87, *p* = 0.028). Years of experience also significantly influenced practices; firefighters with 30–40 years of experience had an aOR of 0.02 (95% CI: 0.001–0.55, *p* = 0.019), and those with more than 40 years of experience had an aOR of 0.011 (95% CI: 2.06–0.594, *p* = 0.027).

## 4. Discussion

Our findings suggest that firefighters’ knowledge and practices scores regarding infection control in the dayroom, in emergency response vehicles, or during emergency runs are generally low. We evaluated and reported on the descriptive characteristics of knowledge and practice scores stratified by firefighter groups (career and volunteers) in the Green River Firefighter Association (GRFA) in Northwestern Kentucky. This region is primarily rural with limited resources but has several volunteer fire departments.

Compared to career firefighters, volunteer firefighters are more likely to have more than 20 years of experience and have higher knowledge and practice scores. For this occupational group, there are limited data in the literature on how years of experience influences knowledge and practice levels. For the allied professions, including other related prehospital emergency response services, studies found experience as one of the major factors that contribute to high practice score and IPC compliance [17,18]. Career firefighters are expected to have received comprehensive training in emergency response including infection control and best practices. Previous studies have found that first responders are often faced with various challenges including working under an emerging threat environment, keeping up to date with growing information, the lack of available training and education, and changes in their daily work responsibilities [4,19,20]. Professional groups like firefighters need to have sustainable strategies to improve knowledge through continuing education that focuses on targeting emerging and reemerging infectious agents, while also including technologies with proven efficiency to protect patient and worker health.

Our data suggest that self-reported practice scores on IPC are higher among volunteer firefighters than among career firefighters. We also found that those who reported to have followed the infection control guidelines did better in practice scores. In a similar study on determining training and education needs pertaining to highly infectious disease preparedness, Le and colleagues found that more than one-third of career firefighters incorrectly marked transmission routes for highly infectious diseases [21]. Le et al. also found discrepancies in self-reports on the existence of highly infectious disease orientation and skills demonstration, employee resources, and PPE policies [21]. It is evident that firefighters are trained to understand the basics of communicable diseases’ transmission and the importance of IPC. While they have the necessary training, some still take risks during the heat of the crisis while they are focused on the job outcomes and sometimes make compromises resulting in mistakes outside of IPC. Career firefighters are often overwhelmed with emergency response duties and naturally will pay more attention to physical and chemical exposures than biologic agents. Additionally, volunteer firefighters may exhibit better practices because their normal day to day job may greatly influence their IPC knowledge and related behaviors [22].

The findings suggest that in the GRFA firefighters who expressed the need to have comprehensive training on PPE selection have the highest knowledge scores compared to those who expressed the need for basic or intermediate training. Although PPE use, compared to engineering or other higher levels of control options for occupational hazards in the workplace, is usually found to be less effective because of its inherent nature of dependence on worker behaviors, it remains a critical control for hazards involving biologic contaminants. Because firefighters respond to complex situations with limited information including potential exposure to body fluids, providing appropriate training on the selection and use of PPE is essential. Training on IPC and IPC-related policies should emphasize self-protection and protection of patients; environmental sanitation in the dayroom, emergency response vehicles, and during runs; and precautionary actions on IPC.

This study has some limitations. Data were collected from career and volunteer firefighters in Northwestern Kentucky, which is rural. Rural areas place high social status on volunteerism, and volunteer fire stations in these regions receive high social and community support. This could be partly explained by 29.1% of volunteer firefighters who responded to the survey having greater than 20 years of work experience (compared to only 16% for career). Information on the primary occupation of volunteer firefighters was not collected. Primary occupation of volunteer firefighters would have helped to control for the influence on primary job-related knowledge and practice behaviors. Additionally, due to the rural setting of the study area, our population does not include firefighters in large metropolitan urban areas of Kentucky where socioeconomic and educational factors are higher. This study does include counties in small metropolitan areas of Kentucky. This makes the results less generalizable to large metropolitan areas of the United States. Despite these limitations, this paper provides essential information on training needs and IPC policies for individual career and volunteer fire departments in rural to small metropolitan areas, the GRFA, and other collaborators.

## 5. Conclusions

This study reveals overall knowledge on infection control and related practice behavior is low among rural firefighters in Northwestern Kentucky. Specifically, our finding suggests that firefighter knowledge and practice on infection control in the dayroom, in emergency response vehicles, or during emergency runs are low. Firefighters who had not received infection control prevention training had significantly lower knowledge scores. Volunteer firefighters exhibited significantly better infection control practices than career firefighters. Firefighters who did not follow the infection control guidelines had significantly worse practices scores. On the other hand, firefighters who expressed the need for comprehensive training on PPE selection appropriate for environmental contaminants also show better practice scores. Considering these results, it will be useful to focus resources on personnel training to improve practice behaviors backed by good foundational knowledge in infection prevention and control. To make this effective and sustainable, there is an urgent need to review rural firefighter infection prevention and control policy.

## Figures and Tables

**Table 1.** Firefighter training needs on PPE selection.

Basic Training (*Needs Training on Any Two of the Following*)	Intermediate Training (*Needs Training on Any Three of the Following*)	Comprehensive Training (*Needs Training on All of the Following*)
How to protect against self-contamination	How to protect against self-contamination	How to protect against self-contamination
Safe removal of disposable gloves	Safe removal of disposable gloves	Safe removal of disposable gloves
How and when PPE should be worn	How and when PPE should be worn	How and when PPE should be worn
What to do in an event of equipment failure	What to do in an event of equipment failure	What to do in an event of equipment failure
		Do not know

**Table 2.** Characteristics of study participants by firefighter status.

	Career (N = 210)	Volunteer (N = 234)	Total (N = 444)	*p*-Value
**Gender**				0.001 ^1^
Male	205 (97.6%)	202 (86.3%)	407 (91.7%)	
Female	5 (2.4%)	32 (13.7%)	37 (8.3%)	
Firefighter groups				0.028 ^1^
Firefighter Response	122 (58.1%)	142 (60.7%)	264 (59.5%)	
Fire Chief (Administrator)	20 (9.5%)	34 (14.5%)	54 (12.2%)	
Emergency Medical Service (EMS)	46 (21.9%)	34 (14.5%)	80 (18.0%)	
Fire instructors and Safety Officers	20 (9.5%)	15 (6.4%)	35 (7.9%)	
Others	2 (1.0%)	9 (3.8%)	11 (2.5%)	
Age in categories				0.001 ^1^
<30 years	44 (21.0%)	65 (27.8%)	109 (24.5%)	
30–40 years	86 (41.0%)	63 (26.9%)	149 (33.6%)	
>40 years	80 (38.1%)	106 (45.3%)	186 (41.9%)	
Years of experience				0.001 ^1^
10 years and below	91 (43.3%)	111 (47.4%)	202 (45.5%)	
11–20 years	85 (40.5%)	55 (23.5%)	140 (31.5%)	
20 years and above	34 (16.2%)	68 (29.1%)	102 (23.0%)	

1 Pearson’s Chi-squared test, statistically significant at *p* < 0.05.

**Table 3.** Achieved scores in the “knowledge” and “practices” dimensions related to firefighter characteristics.

Variable	N (%)	Knowledge Score (%)			Practices Score (%)		
		Mean	SD	*p*-Value	Mean	SD	*p*-Value
**Gender**							
Male	397 (92.1%)	62.5	14.2	0.039 *	68.8	12.0	0.079 *
Female	34 (7.9%)	66.7	11.4		73.0	13.9	
**Age**							
<30 years	105 (24.4%)	63.9	11.8	0.639 ^ꝉ^	70.0	12.1	0.505 ^ꝉ^
30–40 years	145 (33.6%)	62.2	14.1		69.4	12.0	
>40 years	181 (42.0%)	63.1	13.5		68.3	12.7	
**Firefighter status**							
Career firefighter	209 (48.5%)	62.5	13.9	0.639 *	67.4	11.1	0.005
Volunteer firefighter	222 (51.5%)	63.1	14.2		70.6	13.0	
**Years of experience**							
10 years and below	196 (45.5%)	63.5	14.0	0.600 ^ꝉ^	70.9	11.9	0.033 ^ꝉ^
11–20 years	135 (31.3%)	63.0	14.4		67.9	12.6	
20 years and above	100 (23.2%)	61.8	13.3		67.9	12.1	
**Firefighter groups that best describe participants**							
Firefighter Response	264.0 (59.5%)	62.1	13.6	0.015 ^ꝉ^	69.2	11.7	0.943 ^ꝉ^
Fire Chief (Administrator)	54.0 (12.2%)	64.1	14.1		68.8	12.3	
Emergency Medical Service	80.0 (18.0%)	64.2	15.6		69.8	12.6	
Instructor + Safety	35.0 (7.9%)	61.8	14.3		67.4	14.9	
Others	11.0 (2.5%)	71.9	9.1		68.3	13.7	
**If firefighters follow infection prevention guidelines**							
True	416 (98.3%)	62.8	14.0	0.925 *	69.5	11.9	0.012
False	7 (1.7%)	62.3	17.7		58.1	9.9	
**Use of PPE during response to emergency calls**							
True	29 (6.9%)	59.7	14.9	0.173	67.6	9.4	0.395
False	394 (93.1%)	63.1	14.0		69.4	12.1	
**Types of PPE wore during COVID-19 responses**							
Gloves	2 (0.5%)	54.5	0.0	0.434 ^ꝉ^	56.6	23.5	0.313 ^ꝉ^
Isolation gown	5 (1.2%)	59.1	15.9		76.7	16.7	
Facemask (cloth face covering)	24 (5.6%)	59.9	13.8		69.2	10.4	
Respirator (N-95 or higher-level respirator)	396 (91.9%)	63.4	13.9		69.3	11.8	
Personal eyeglasses/goggles	3 (0.7%)	54.5	9.0		71.1	7.6	
Eye protection (face shields)	1 (0.2%)	54.5	0.0		60.0	9.4	
**If firefighter responded to COVID-19 call**							
Yes	328 (76.1%)	63.6	13.8	0.059 *	69.2	11.7	0.906 *
No	103 (23.9%)	60.7	14.6		69.3	12.8	
**If firefighter responded to COVID-19 calls while on the job**							
Yes, I had one/multiple encounters while on the job	324 (76.6%)	63.6	14.0	0.056	69.5	11.6	0.407
No, I have not had an encounter while on the job	99 (23.4%)	60.8	14.2		68.4	12.9	
**If firefighter is trained on infection prevention**							
Yes	341 (80.6%)	63.7	13.4	0.012 *	69.6	11.6	0.140
No	82 (19.4%)	59.7	15.9		67.6	12.9	
**Topics to be addressed when training firefighters on PPE selection**							
Basic training on use and selection	75(17.7%)	57.6	12.5	<0.001 ^ꝉ^	66.7	13.3	0.110 ^ꝉ^
Intermediate training	39 (9.2%)	59.7	13.9		69.8	12.3	
Comprehensive training	309 (73.0%)	64.8	14.0		70.2	11.3	
**If participants tested positive for COVID-19**							
Yes	135 (31.3%)	64.2	14.2	0.170 *	70.1	11.0	0.305 *
No	296 (68.7%)	62.2	14.0		68.9	12.1	

Statistically significant *p*-values are indicated in bold;

* unpaired *t*-test, equal variances;

ꝉ analysis of variance;

PPE, personal protective equipment.

**Table 4.** Association between firefighter knowledge and factors related to infection control.

	95% aOR CI						

Variables	β	SE	aOR	Lower	Upper	z	*p*-Value
(Intercept)	63.83	1.60	5.24	2.28	1.20	39.93	<0.001
Firefighter status							
Volunteer firefighter	1.79	1.51	6.00	0.31	115.12	1.19	0.235
Career firefighter (Ref)							
Gender							
Female	4.19	2.55	66.22	0.44	9895.03	1.64	0.101
Male (Ref)							
Participants’ age							
<30 years (Ref)							
30–40 years	−2.61	1.78	0.07	0.002	2.39	−1.46	0.143
>40 years	−2.00	1.75	0.13	0.004	4.13	−1.14	0.252
Response calls for COVID-19							
No	−1.43	2.19	0.24	0.003	17.47	−0.65	0.515
Yes (Ref)							
Call on a job during COVID-19							
No	−1.20	2.24	0.30	0.004	24.07	−0.53	0.591
Yes (Ref)							
Trained on IP							
No	−3.65	1.79	0.02	7.78	0.86	−2.04	0.042
Yes (Ref)							
Specific group that best describes them							
Fire Chief (Administrator)	1.83	2.21	6.22	0.082	470.37	0.82	0.408
Emergency Medical Service	1.57	1.83	4.81	0.13	172.12	0.86	0.390
Instructor, Safety and Others	1.81	2.25	6.12	0.07	507.24	0.80	0.422
Firefighter Response (Ref)							

**Table 5.** Association between firefighter practices and factors related to infection control.

	95% aOR CI						

Variables	β	SE	aOR	Lower	Upper	z	*p*-Value
(Intercept)	63.89	2.50	5.61	4.16	7.55	25.54	<0 .001
Firefighter status							
Volunteer firefighter	4.52	1.21	92.05	8.59	986.25	3.73	<0 .001
Career firefighter (Ref)							
Gender							
Female	2.44	2.07	11.41	0.19	656.48	1.17	0.240
Male (Ref)							
Age							
<30 years (Ref)							
30–40 years	−0.11	1.61	0.89	0.03	21.02	−0.07	0.944
>40 years	1.25	2.00	3.49	0.07	175.77	0.62	0.531
If firefighter followed infection control guidelines							
False	−9.27	4.46	9.46	1.53	0.58	−2.07	0.038
True (Ref)							
Topics to be addressed when training firefighters on PPE selection							
Basic training (Ref)							
Intermediate training	2.64	2.28	14.000	0.160	1221.805	1.1574	0.248
Comprehensive training	3.41	1.54	30.223	1.471	620.877	2.2103	0.028
If firefighter is trained on infection control							
No	−2.37	1.47	0.094	0.005	1.666	−1.6125	0.108
Yes (Ref)							
Years of experience							
<10 years (Ref)							
30–40 years	−3.64	1.55	0.026	0.001	0.550	−2.3461	0.019
>40 years	−4.51	2.03	0.011	2.06	0.594	−2.2166	0.027

## Data Availability

Data will be available upon formal request to any of the authors.

## Appendix A

**Table A1.** Scoring scheme for the fact-based quantifiable questions of KP survey. By default, a score point was added when a response was selected, except when there are data missing or not selected.

Type	Question	Response	Score	Total
Select best answer	An exposure incident refers to the contact of intact skin with blood or other potentially infectious material.	[1] = True [2] = False [3] = Don’t know	1 0 0	1
Select best answer	Post-exposure evaluation and follow-up must be provided on-site.	[1] = True [2] = False [3] = Don’t know	1 0 0	1
Select best answer	Any exposure incident should be reported through the channels outlined in the fire department plan.	[1] = True [2] = False [3] = Don’t know	0 1 0	1
Select best answer	An exposed staff person is responsible for paying for any evaluation or follow-up resulting from the exposure incident.	[1] = True [2] = False [3] = Don’t know	1 0 0	1
Select best answer	Which of the following is the best control method to prevent exposure to bloodborne pathogens?	[1] = Sharp containers [2] = Hepatitis B Vaccination [3] = Training on how to handle soiled linens [4] = Retractable and self-sheathing needles [5] = Don’t know	1 1 1 1	1
Select best answer	It is best practice to start with the dirtiest surfaces when cleaning and disinfecting in a training area/dayroom or emergency response vehicle.	[1] = True [2] = False [3] = Don’t know	0 1 0	1
Select best answer	How do you know if the surfaces are cleaned?	[1] = Periodic culturing of environmental surfaces is recommended to ensure that surfaces have been cleane [2] = Fluorescent markers are a good way to identify surfaces that were missed during cleaning [3] = Visual assessment is the most accurate method for determining whether a surface has been cleaned and disinfected. [4] = Don’t know	0 1 0 0	1
Multiple responses	Which of the following surfaces should be cleaned and disinfected on a daily basis?	[1] = Training area/Dayroom [2] = Emergency response vehicles [3] = Personal Operating Vehicles [4] = Bathrooms [5] = Radios	1 1 1 1 1	5
Maximum total score knowledge on exposure incident control				12
Select best answer	How frequent do you clean the surfaces?	[1] = Daily [2] = Weekly [3] = Monthly	3 2 1	3
Multiple responses	Before performing wound care, if your hands are not visibly dirty, which method of hand cleaning would you prefer?	[1] Using an alcohol-based hand rub [2] = Washing hands with soap and water, followed immediately by use of alcohol-based hand rub [3] = None. Since gloves should be worn when performing patient transport, hand hygiene is not necessary [4] = None of the above. [5] = Don’t know	1 1 0 0 0	2
Multiple responses	When is it essential to perform hand hygiene?	[1] = Before meals [2] = Before patient transport [3] = After patient transport [4] = Before using PPE [5] = After using PPE [6] = After contact with blood or blood fluids [7] = Don’t know	1 1 1 1 1 1 0	6
Select best answer	Cleaning and disinfection should proceed from high areas to low areas.	[1] = True [2] = False [3] = Don’t know	1 0 0	1
Select best answer	Cleaning and disinfection should proceed in a consistent pattern.	[1] = True [2] = False [3] = Don’t know	1 0 0	1
Select best answer	Multiple cleaning cloths are required to clean a single room or workstation.	[1] = True [2] = False [3] = Don’t know	1 0 0	1
Select best answer	Pathogens are spread by contact with contaminated surfaces.	[1] = True [2] = False [3] = Don’t know	1 0 0	
Select best answer	Occupying a dayroom with or after a colleague who is infected with a pathogen places you at high risk for infection.	[1] = True [2] = False [3] = Don’t know	1 0 0	1
Select best answer	Extensive contact (i.e., longer than 2 to 3 min) is necessary for a staff member’s hands to become contaminated with pathogens from environmental surfaces.	[1] = True [2] = False [3] = Don’t know	0 1 0	1
Select best answer	Few healthcare pathogens can survive for more than an hour on dry surfaces.	[1] = True [2] = False [3] = Don’t know	0 1 0	1
Maximum total score infection control practice				18
